# Cartilage decisively shapes the glenoid concavity and contributes significantly to shoulder stability

**DOI:** 10.1007/s00167-022-06968-7

**Published:** 2022-04-17

**Authors:** F. Souleiman, I. Zderic, T. Pastor, P. Varga, T. Helfen, G. Richards, B. Gueorguiev, J. Theopold, G. Osterhoff, P. Hepp

**Affiliations:** 1 Department of Orthopaedics, Trauma and Plastic Surgery, University Hospital Leipzig, Leipzig, Germany; 2 AO Research Institute Davos, Davos, Switzerland; 3 Department of Orthopaedic and Trauma Surgery, Lucerne Cantonal Hospital, Lucerne, Switzerland; 4 Department of Orthopaedics and Trauma Surgery, Musculoskeletal University Center Munich (MUM), University Hospital, LMU Munich, Munich, Germany

**Keywords:** Shoulder stability ratio, Shoulder dislocation, Cartilage, Glenoid concavity, Shoulder instability, GLAD lesion

## Abstract

**Purpose:**

Glenohumeral joint injuries frequently result in shoulder instability. However, the biomechanical effect of cartilage loss on shoulder stability remains unknown. The aim of the current study was to investigate biomechanically the effect of two severity stages of cartilage loss in different dislocation directions on shoulder stability.

**Methods:**

Joint dislocation was provoked in 11 human cadaveric glenoids for 7 different directions between 3 o'clock (anterior) and 9 o'clock (posterior). Shoulder stability ratio (SSR) and concavity gradient were assessed in three states: intact, 3 mm and 6 mm simulated cartilage loss. The influence of cartilage loss on SSR and concavity gradient was statistically evaluated.

**Results:**

Both SSR and concavity gradient decreased significantly between intact state and 6 mm cartilage loss in every dislocation direction (*p* ≤ 0.038), except concavity gradient in 4 o'clock direction. Thereby, anterior–inferior dislocation directions were associated with the highest decrease in both SSR and concavity gradient of up to 59.0% and 49.4%, respectively, being significantly bigger for SSR compared with all other dislocation directions (*p *≤ 0.040). Correlations between concavity gradient and SSR for pooled dislocation directions were significant in each separate specimen's state (*p *< 0.001).

**Conclusion:**

From a biomechanical perspective, articular cartilage of the glenoid contributes significantly to the concavity gradient, correlating strongly with the associated loss in glenohumeral joint stability. The biggest effect of cartilage loss is observed in the most frequently occurring anterior–inferior dislocation directions, suggesting that surgical interventions to restore cartilage's surface and concavity should be considered for recurrent shoulder dislocations in presence of cartilage loss.

**Supplementary Information:**

The online version contains supplementary material available at 10.1007/s00167-022-06968-7.

## Introduction

Glenohumeral bone and cartilage lesions are common injuries associated with recurrent shoulder instabilities [8, 20, 21, 36]. The latter are present in either acute or chronic form [8, 14]. Cartilage lesions are diagnosed in up to 79% of cases associated with shoulder instabilities [8, 14, 28]. Dynamic and static shoulder stabilisers exist as the shoulder is the joint with the highest range of motion and a mismatch between the humeral and glenoid joint surface at a ratio of 4:1 [15]. Static stabilisers include the labrum, ligaments, and the cartilage–bone morphology of the glenoid. The latter has a typical concave shape with the lowest point located centrally and a high glenoid rim [15]. Concavity compression is a biomechanical key factor for joint stability especially in the range of motion with maximum ligament and capsular laxity [35]. The labrum of the glenoid adds up to 50% depth to glenoid concavity. A layer of cartilage is deposited on the glenoid bone, increasing in thickness from the centre to the outer rim [15]. This cartilage layer increases the concavity gradient and concavity compression in addition to the concave shape of the bone [33]. In case of recurrent dislocations, higher grade loss of cartilage is reported [14]. Since anterior shoulder dislocations are the most common ones, the so called glenolabral articular disruption (GLAD lesion) is of particular importance [23, 26, 27, 30, 34]. These lesions are characterised by labral tears following a shoulder dislocation event. Deep fibres, interwoven with the articular cartilage, cause various types of avulsion type cartilage loss [27].

The effect of labrum and bone defects on shoulder stability has been extensively studied [16, 20, 21, 35]. However, the cartilage contribution to the concavity gradient and the corresponding biomechanical influence of a cartilage defect or cartilage loss on shoulder stability ratio (SSR) in dependence of dislocation direction remain unclear. In absence of knowledge about such association, it is unclear which cartilage lesions require surgical treatment.

Therefore, the aim of the present study was to evaluate the biomechanical effect of cartilage loss on SSR in different dislocation directions ranging from anterior to posterior. It was hypothesised that the degree of cartilage loss has a significant effect on shoulder instability requiring reconstructive cartilage repair.

## Materials and methods

### Specimens and preparation

**Fig. 1:**
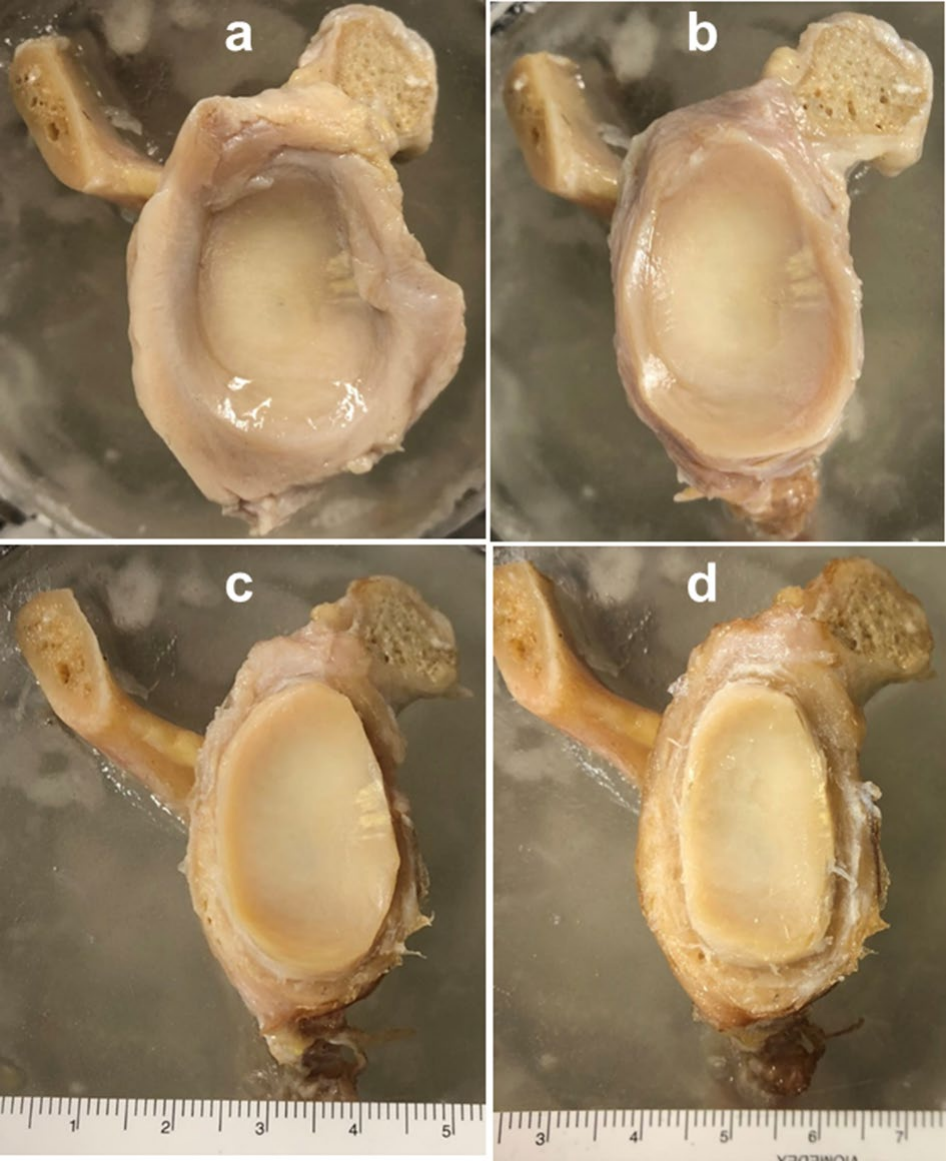
Exemplified photographic documentation of the three different specimen's states. All preparations were performed after specimen’s embedding in PMMA: **a** native glenoid with parts of the labrum; **b** intact state after labrum removal; **c** 3 mm state of cartilage loss; **d** 6 mm state of cartilage loss

Six right and five left fresh–frozen (−20℃) human cadaveric scapulae from six male and five female donors aged 54.3 years on average (range 24–75 years) with no visible preexisting pathology, trauma or surgery were used. All donors gave their informed consent inherent within the donation of the anatomical gift statement during their lifetime. The specimens were thawed at room temperature 24 h before preparation and embedded at the level of glenoid neck in a polymethylmethacrylate (PMMA; SCS-Beracryl, Suter-Kunststoffe AG, Fraubrunnen, Switzerland) cylinder, with the glenoid plane oriented perpendicular to the cylinder axis. All soft tissues, including the labrum were removed to focus on the effects of osteochondral integrity on glenohumeral stability. The acromion and the coracoid were removed from the scapula to ensure a humeral movement without restriction. Biomechanical testing of each glenoid was performed in the following three states: intact, 3 mm and 6 mm cartilage loss (Fig. 1). A digital calliper (Futuro, Switzerland) with an accuracy of 0.01 mm was used for marking prior to cutting the cartilage with a scalpel for simulation of its loss. The marks were placed circularly, starting from the glenoid rim.

### Image processing

**Fig. 2:**
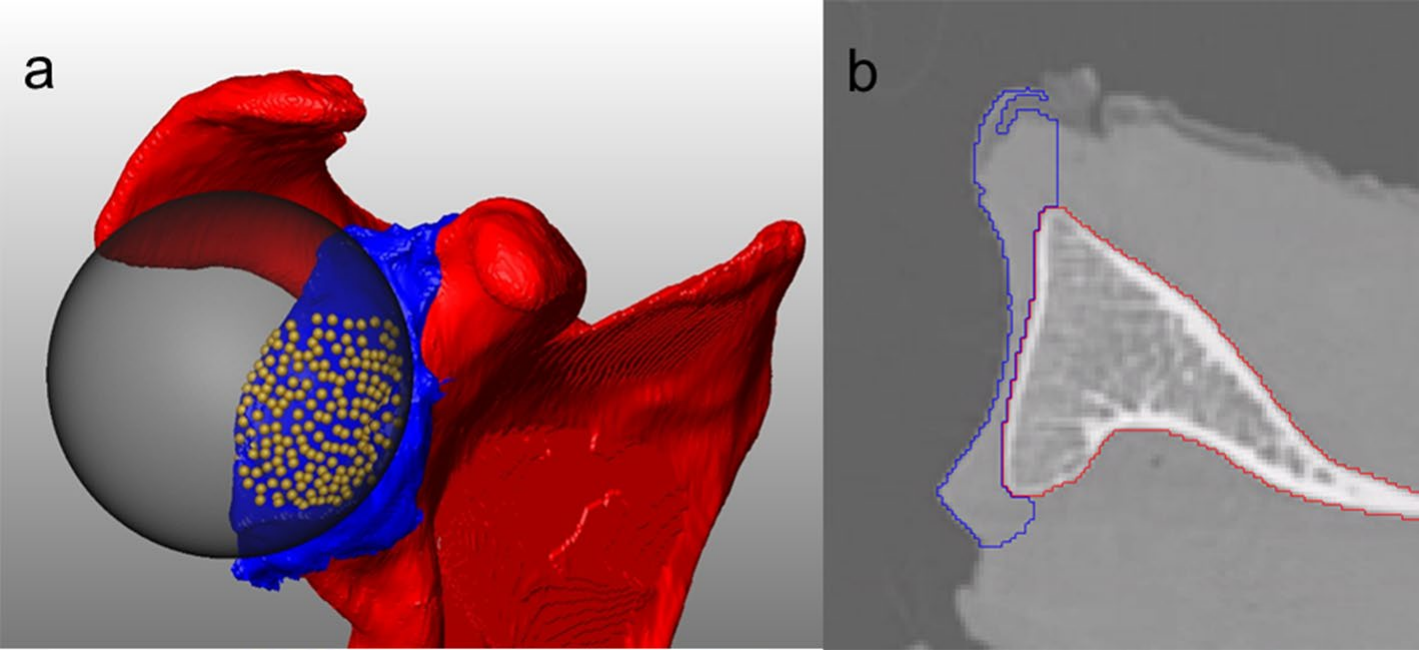
**a** Exemplified 3D segmented model of a scapula. Red and blue areas represent bony scapula and overlying cartilage, respectively. An individually calculated best fitting sphere (dark-grey transparent) is generated using anatomical landmarks on the cartilage surface (yellow dots). **b** The corresponding axial CT slice illustrates how the thickness of the cartilage layer increases from its centre to the outer rim and ends in the labrum (blue)

Prior to biomechanical testing all specimens underwent computed tomography (CT) scanning (Revolution EVO, GE Medical Systems (Schweiz) AG, Switzerland, slice thickness: 0.625 mm, energy: 120 kVp, X-ray tube current: 200 mA, convolution kernel: BONE) for morphological analysis of the glenoids in terms of radius, width, length and depth. Using an image processing software package (Amira, Version 2020.3, Berlin, Germany), separate three-dimensional (3D) models of the scapula and the overlying cartilage layer were created (Fig. 2). To define the diameter of the simulated humeral head used for biomechanical testing, the best fitting sphere was processed by landmarking the glenoid surface of each specimen separately (accuracy: 0.1 mm).

### Biomechanical testing

Biomechanical testing was performed on a material testing system (Acumen 3, MTS Systems Corp., Eden Prairie, MN, USA) equipped with a 250 N load cell. The equipment was calibrated according to DIN EN ISO 7500, fulfilling the requirements for a class 1 accuracy level within the range of ±1% for both transducer (displacement) and load cell (force). The test setup was designed to simulate shoulder dislocations in seven different directions, based on the ‘rocking horse’ experiment for assessment of glenoid loosening defined by ASTM F2028. The glenoid was mounted with its plane oriented vertically to the machine base via an interconnected xy-table. A metal fixture holding the PMMA embedding allowed for accurate in-plane rotation of the glenoid. A rotary knob was used to block the rotation once the desired position was set. Markers were attached to the machine transducer and the xy-table for motion tracking.

To avoid possible effects of cartilage lesions at the humeral side, dislocations were induced with custom made metal spheres attached to the machine transducer and simulating humeral heads. Spheres with diameters of 37, 39, 41, 43, 45, 47, 49, 51, and 53 mm were fabricated.

A constant compression force of 50 N was applied between the glenoid and the metal sphere horizontally and perpendicular to the glenoid plane via an attached weight, pulling on the xy-table via a cable-and-pulley system [35]. Vaseline was used between the artificial humeral head and the glenoid to minimise the effect of friction.

For each dislocation direction, the initial position of the metal sphere relative to the glenoid was defined in intact specimen's state. By applying 50 N joint compression, the sphere naturally dropped into the glenoid groove. To accurately adjust it in the same starting position for each separate dislocation test of the specimen over the two different states of cartilage loss, a virtual coordinate system was defined. The vertical position of the sphere corresponded to the z coordinate (machine transducer's axis) and was read out from the machine software. Once the initial position was defined, the xy-table was constrained via adjusting screws in y direction, defined by the horizontal movement in the glenoid plane. An interconnected measurement calliper served as indicator of the initial position along this direction.

Each specimen was rotated in 30° in-plane steps to test seven dislocation directions, defined for simplification according to an hour hand of a dial, with the anterior direction being 3 o'clock, the inferior direction—6 o'clock, and the posterior direction—9 o'clock one. The rest 30° step directions were defined as 4, 5, 7 and 8 o'clock ones. For each tested dislocation direction, the glenoid was rotated so that the respective hour hand was oriented vertically and pointed from its centre downwards, aligned with the z axis of the coordinate system.

Testing commenced in 3 o'clock and ended in 9 o'clock direction. A shoulder dislocation was provoked by the machine transducer, actuated in four subsequent ramps performed in displacement control at a rate of 1 mm/s. The test began with the actuator positioned 3 mm above the initial sphere position and ended 17 mm below it. The movement of 20 mm ensured that the metal sphere slipped over the glenoid rim, simulating shoulder dislocation. Throughout the test, the x direction of the xy-table was left unconstrained, whereas the y direction was blocked to exclude biased influence of any horizontal movements in the glenoid plane. All measurements were repeated for all specimen’s states—intact, 3 mm and 6 mm cartilage loss.

### Data acquisition and analysis

Machine data in terms of axial displacement and axial force were continuously recorded from the machine actuators at 128 Hz. The peak reaction forces recorded during the last three ramps of each dislocation test were averaged and defined as maximum reaction force Fmax. The SSR represents the relationship between the maximum force required to dislocate the humeral head out of the glenoid and the forces centring the humeral head [4, 16, 20, 35]. Accordingly, it was defined as the percentage ratio between Fmax and the 50 N constant glenoid compression force between glenoid and sphere (Fcons):

$${\text{SSR}} = \frac{{F_{\max } }}{{F_{{{\text{cons}}}} }} \times 100\% .$$

The coordinates of the markers, attached to the xy-table and the transducer, were continuously recorded throughout the dislocation tests using a stereographic optical camera system (Aramis SRX, GOM GmbH, Braunschweig, Germany) operating at 12 megapixel resolution and 0.004 mm maximum acceptance error. Based on the motion tracking data, the relative movements of the xy-table in x direction relative to the sphere movement in z direction were calculated. The concavity gradient was calculated as maximum slope of the curve defined by these two movements.

### Statistical analysis

Statistical analysis upon the parameters of interest Fmax, SSR and concavity gradient was performed with SPSS software package (V.27, IBM, NY, USA). Normal distribution of the data was screened and confirmed with Shapiro–Wilk test for all dislocation directions and specimen's states separately. Descriptive data are presented in terms of mean and standard deviation (SD). The gradual evolvement of Fmax, SSR and concavity gradient over all specimens’ states was screened with General Linear Model Repeated Measures with Bonferroni post hoc tests for multiple comparisons in each separate dislocation direction. Next, the relative loss of SSR and concavity gradient at 3 mm and 6 mm cartilage loss with respect to the intact state, and 6 mm cartilage loss relative to the 3 mm state were computed for each specimen and dislocation direction separately. Based on this, the influence of dislocation direction on the relative loss of both SSR and concavity gradient were analysed with Paired-Samples T-test. Finally, Pearson's correlation coefficients (PCC) were calculated over the three specimen’s states to measure the linear correlation between Fmax/SSR and concavity gradient for each separate dislocation direction, as well as for the pooled data over all dislocation directions. Level of significance was set to 0.05 for all statistical tests. A priori power analysis resulted in minimum of 10 specimens required for statistical power of 0.8 under the presumption that the standard deviation related to each specimen’s state does not exceed 80% of the difference in the corresponding mean values between two states.

## Results

**Fig. 3:**
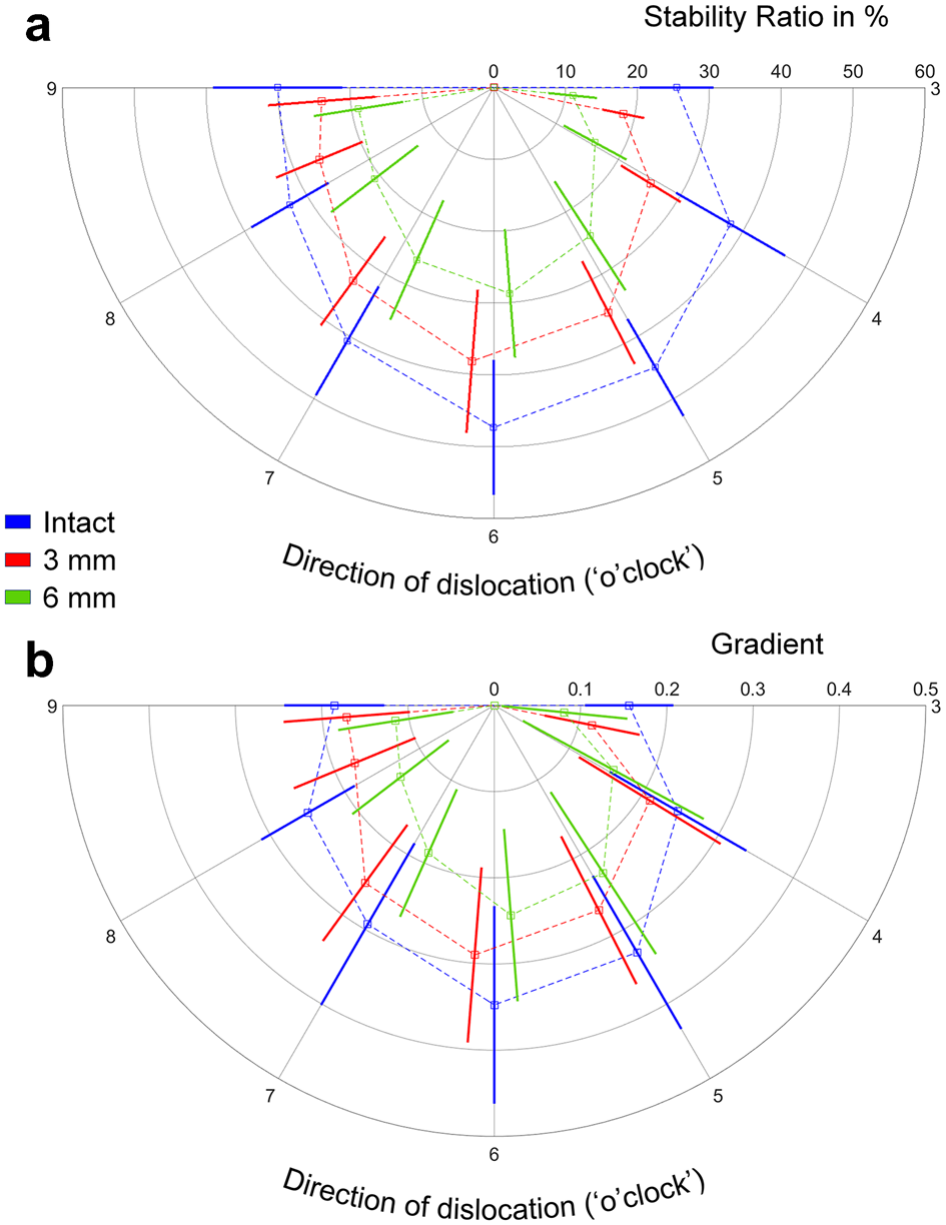
Radial plot showing **a** SSR (%) and **b** concavity gradient (unitless) for each dislocation direction at different specimen’s states in terms of mean and SD

SSR decreased significantly within the course of cartilage loss for each dislocation direction (intact to 3 mm: *p* ≤ 0.004; intact to 6 mm: *p* ≤ 0.001; 3 mm to 6 mm: *p* ≤ 0.002) except between the intact and 3 mm states in 9 o'clock dislocation direction (Table 1, Fig. 3).

**Table 1:** Absolute and percentage loss for SSR and concavity gradients between intact and 3 mm, and between intact and 6 mm states, with corresponding p-values

Direction (o'clock)	Differences between states of cartilage loss					
	Intact to 3 mm			Intact to 6 mm		
	Absolute loss	Percentage loss (%)	*p* value	Absolute loss	Percentage loss (%)	*p* value
SSR						
3	7.1	30.0	<0.001	14.4	58.7	<0.001
4	12.4	31.5	<0.001	22	57.2	<0.001
5	9.8	21.9	<0.001	20.4	44.9	<0.001
6	9.1	19.4	0.001	18.6	38.9	<0.001
7	7.4	17.6	0.002	14.4	35.3	<0.001
8	6.5	19.6	0.004	11.8	36.8	<0.001
9	6.1	17.9	n.s.	11	35.5	0.001
Concavity gradients						
3	0.05	28.5	0.027	0.08	49.4	0.038
4	0.03	20.4	n.s.	0.10	38.5	n.s.
5	0.07	20.8	<0.001	0.10	29.3	0.033
6	0.06	17.0	<0.001	0.13	35.2	0.002
7	0.05	15.0	0.013	0.12	39.2	<0.001
8	0.05	23.7	0.016	0.09	38.3	0.001
9	0.01	2.9	n.s.	0.06	29.0	<0.001

Between intact and 6 mm states, the loss of SSR was significantly higher in 3 and 4 o'clock dislocation directions compared with all other dislocation directions (*p* ≤ 0.04) with a mean maximum loss of 58.7% in 3 o'clock direction.

Concavity gradient decreased within the course of cartilage loss for each dislocation direction, being significant between intact and 3 mm states in 3, 5, 6, 7 and 8 o'clock directions (*p* ≤ 0.027), and between intact and 6 mm states in 3, 5, 6, 7, 8 and 9 o'clock directions (*p* ≤ 0.038 Table 1, Fig. 3).

Between intact and 6 mm states, the loss of concavity gradient was homogenously distributed among all dislocation directions with no significant differences between them, and with a mean maximum loss of 49.4% in 3 o'clock direction.

Finally, the correlations between SSR and concavity gradient were significant for pooled dislocation directions in each separate specimen’s state (intact: PCC = 0.894, *p* < 0.001; 3 mm: PCC = 0.904, *p* < 0.001; 6 mm: PCC = 0.703, *p* < 0.001).

## Discussion

The most important finding of the present study was that cartilage contributes considerably to the glenoidal concavity, correlating with the associated glenohumeral joint stability. The severity of cartilage loss has a significant effect on shoulder stability and the strength of this effect depends on the direction of dislocation.

One possible explanation for the decreasing stability during cartilage loss is that the concavity of the glenoid is essentially built up by the cartilage [33]. This was confirmed by the fact that the concavity gradient decreased with each further state of induced cartilage loss.

Another important finding was that the cartilage layer demonstrated different concavity gradients in different dislocation directions. Thus, the degrees of cartilage loss resulted in stronger decreases in both concavity gradient and SSR for anterior–inferior dislocations compared with the other directions.

The biomechanical influence of the labrum and bone concavity on SSR in the glenoid has been investigated in previous work [2, 16, 20, 35]. Clinical studies suggested that a more frequent number of dislocations are associated with larger cartilage defects [14, 28]. In combination with the findings of the current study this suggests that cartilage loss leads to instability, higher rate of dislocations and thus to further cartilage loss.

In particular, active athletes with a high shoulder demand have an increased risk of suffering from cartilage lesions [7, 8, 27, 28]. Recent studies have reported that the latter are present in up to 78.6% of patients with shoulder instability [14, 24, 28]. A systematic review concluded that cartilage defects without bony lesions are rare or often overlooked [28]. Radiological studies could show that the imaging conditions are crucial to identify such lesions [30]. Tian et al. were able to detect increased labroligamentous lesions in abduction and external rotation [30]. Principally, an acute cartilage loss must be differentiated from a chronic one [28, 37]. The latter is associated with overuse where shoulder stabilisers and activities can adapt [6, 28, 29]. Acute cartilage lesions are usually associated with adequate trauma [28]. Based on the outcomes from this study, surgeons need to be more sensitive to cartilage lesions as a possible cause of recurrent shoulder instability regardless of an acute or chronic aetiology.

Special attention should be paid to GLAD lesions, where the bony stabilisers remain intact [23, 27]. These can follow adequate trauma but can occur more frequently after recurrent dislocations [10, 24, 25]. In contact, for athletes, such as rugby players, GLAD lesions were observed in relevant rates of up to 15% [12, 22]. Such lesions are predominantly reported for anterior–inferior directions [26, 27, 34]. The present study could demonstrate that cartilage loss for anterior–inferior dislocation directions were associated with the highest loss of both SSR and concavity gradient compared to other directions. Accordingly, anterior–inferior GLAD lesions are biomechanically more relevant than those cartilage defects affecting other dislocation directions.

Treatment of shoulder instability in glenoids with osseous or cartilage loss is challenging and has a higher incidence of recurrent dislocation [8, 14]. The integrity of the glenoid is a key factor for the postoperative outcome [1, 3, 5, 13, 14, 18]. Pogorzelski et al. reported higher failure rates for GLAD-like lesions [26]. The current study provides a possible explanation for this from a biomechanical point of view. Surgical treatment of cartilage and GLAD-like lesions could be considered as a pragmatic approach. Possible surgical treatments include microfracturing, labral coverage and Autologous Matrix-Induced Chondrogenesis (AMIC^®^) [9, 17, 19, 25, 31, 32]. In addition, ex vivo cartilage cultivation and tissue engineering via 3D printing could be future approaches.

The present study has some limitations similar to those inherent to all human cadaveric biomechanical investigations using a limited number of specimens. In addition, the setup was restricted to testing the static stabilisers bone and cartilage. To minimise complexity, dynamic stabilisers of the shoulder were not simulated. A metal sphere was used instead of a humeral head, which could have had an influence on friction. The study was performed on glenoids from donors of advanced age. It can be assumed that the thicker cartilage layer in affected young patients with in vivo shoulder dislocations would have an even higher biomechanical effect on SSR compared with the used cadavers. Furthermore, the glenoid was embedded horizontally. A retroversion, occurring clinically, was not simulated [11]. It can be assumed that in case of retroversion an anterior cartilage defect would lead to an even higher loss of concavity and stability in vivo. Furthermore, it has been demonstrated that the anterior–inferior direction of dislocation is more vulnerable to cartilage defects compared to the other dislocation directions. The advanced donor age must be considered when interpreting the results because increased posterior cartilage loss over the lifetime is conceivable due to possible glenoid retroversion.

A major advantage of this study is the measurement of concavity by motion tracking and its coupling with the necessary dislocation force. In addition, the implementation of a coordinate system allowed high consistency of the measurements when investigating the different states of cartilage loss. Consequently, very precise and accurate measurements could be performed.

This basic biomechanical study investigates the involvement of cartilage on glenoid concavity using two selected resections. In general, cartilage loss in shoulder instability is clinically poorly studied. The presented study is not able to provide a cutoff value of cartilage loss at which surgical intervention is necessary. This needs to be further investigated in clinical studies.

## Conclusion

From a biomechanical perspective, articular cartilage of the glenoid contributes significantly to the concavity gradient, correlating strongly with the associated loss in glenohumeral joint stability. The biggest effect of cartilage loss is observed in the most frequently occurring anterior–inferior dislocation directions, suggesting that surgical interventions to restore cartilage’s congruent surface and concavity should be considered for recurrent shoulder dislocations in presence of cartilage loss.

## Supplementary Information

Below is the link to the electronic supplementary material.

Supplementary file1 (DOCX 19 kb)

## Abbreviations

3D: Three-dimensional
AMIC: Autologous matrix-induced chondrogenesis
ASTM: American society for testing materials
CT: Computed tomography
Fcons: Constant compression force of 50 N between artificial humeral head and glenoid
Fmax: Maximum force required to dislocate the humeral head out of the glenoid
GLAD: Glenolabral articular disruption
PCC: Pearson correlation coefficient
PMMA: Polymethylmethacrylate
SSR: Shoulder stability ratio
