# Supplementary material for: Cartilage decisively shapes the glenoid concavity and contributes significantly to shoulder stability
Source: Knee Surg Sports Traumatol Arthrosc. 2022 Apr 17;30(11):3626–33. doi: 10.1007/s00167-022-06968-7 (PMC9568486; doi:10.1007/s00167-022-06968-7)
Supplement: Supplementary file 1 — Supplementary file1 (DOCX 19 kb) [file 167_2022_6968_MOESM1_ESM.docx]

**Additional files**

Article title: Cartilage decisively shaping the glenoid concavity and contributing significantly to shoulder stability

Journal: Knee Surgery, Sports Traumatology, Arthroscopy (KSSTA)

Authors:

F. Souleiman^1,2^ (ORCID: 0000-0003-1087-7503)
I. Zderic^2^ (ORCID: 0000-0003-0484-887X)
T. Pastor^2,3^ (ORCID: 0000-0003-4357-236X)
P. Varga^2^ (ORCID: 0000-0003-2738-6436)
T. Helfen^4^ **(ORCID: 0000-0002-9462-7976)**
G. Richards^2^ (ORCID: 0000-0002-7778-2480)
B. Gueorguiev^2^ (ORCID: 0000-0001-9795-115X)
J. Theopold^1^ (ORCID: 0000-0003-2884-8030)
G. Osterhoff^1^ (ORCID: 0000-0001-5051-0998)
P. Hepp^1^ (ORCID: 0000-0002-6510-2943)

**Corresponding author**
Firas Souleiman
Department of Orthopedics, Trauma and Plastic Surgery, University of Leipzig

Liebigstraße 20, 04103 Leipzig, Germany
Phone: + 49-341-97-23200; Fax: + 49-0341-97-23209

Email: firas.souleiman@medizin.uni-leipzig.de

Table 2: Fmax, SSR (%) and concavity gradient presented for each cutting stage and dislocation direction separately in terms of mean and SD

| *Dislocation (o'clock)* | *Fmax in N/ SSR in %* | | | *Concavity gradient* | | |
| --- | --- | --- | --- | --- | --- | --- |
|  | *intact* | *3mm* | *6mm* | *intact* | *3mm* | *6mm* |
| 3 | 12.7 ± 2.6/  25.4 ± 5.2% | 9.2 ± 1.5/  18.4 ± 3.0% | 5.5 ± 5.1/  11.0 ± 10.2% | 0.17 ± 0.04 | 0.12 ± 0.06 | 0.09 ± 0.08 |
| 4 | 19.0 ± 4.4/  38.0 ± 8.8% | 12.8 ± 2.4/  25.6 ± 4.8% | 8.0 ± 2.5/  16.0 ± 5.0% | 0.26 ± 0.07 | 0.23 ± 0.09 | 0.16 ± 0.12 |
| 5 | 22.5 ± 3.9/  45.0 ± 7.8% | 17.6 ± 4.0/  35.1 ± 8.0% | 12.3 ± 4.5/  24.6 ± 9.1% | 0.35 ± 0.08 | 0.28 ± 0.08 | 0.25 ± 0.11 |
| 6 | 23.7 ± 4.7/  47.3 ± 9.4% | 19.1 ± 5.0/  38.2 ± 10.0% | 14.4 ± 4.5/  28.7 ± 9.0% | 0.37 ± 0.09 | 0.31 ± 0.09 | 0.24 ± 0.11 |
| 7 | 20.4 ± 4.4/  40.7 ± 8.8% | 16.6 ± 3.8/  33.3 ± 7.6% | 13.1 ± 4.5/  26.3 ± 9.1% | 0.32 ± 0.08 | 0.27 ± 0.08 | 0.20 ± 0.08 |
| 8 | 16.4 ± 3.1/  32.7 ± 6.2% | 13.1 ± 3.3/  26.2 ± 6.5% | 10.4 ± 3.8/  20.9 ± 7.6% | 0.24 ± 0.06 | 0.19 ± 0.07 | 0.15 ± 0.06 |
| 9 | 15.0 ± 4.5/  30.1 ± 9.0% | 12.0 ± 3.7/  24.0 ± 7.5% | 9.5 ± 3.1/  19.0 ± 6.3% | 0.19 ± 0.06 | 0.18 ± 0.07 | 0.13 ± 0.07 |
